# The Benefits of Probiotics on Oral Health: Systematic Review of the Literature

**DOI:** 10.3390/ph16091313

**Published:** 2023-09-16

**Authors:** Francesco Inchingolo, Angelo Michele Inchingolo, Giuseppina Malcangi, Nicole De Leonardis, Roberta Sardano, Carmela Pezzolla, Elisabetta de Ruvo, Daniela Di Venere, Andrea Palermo, Alessio Danilo Inchingolo, Alberto Corriero, Gianna Dipalma

**Affiliations:** 1 Department of Interdisciplinary Medicine, University of Bari “Aldo Moro”, 70124 Bari, Italy; angeloinchingolo@gmail.com (A.M.I.); giuseppinamalcangi@libero.it (G.M.); nicoledeleonardis@outlook.it (N.D.L.); robertasardano@gmail.com (R.S.); c.pezzolla3@studenti.uniba.it (C.P.); studio.deruvo@libero.it (E.d.R.); daniela.divenere@uniba.it (D.D.V.); ad.inchingolo@libero.it (A.D.I.); alberto.corriero@gmail.com (A.C.); giannadipalma@tiscali.it (G.D.); 2 College of Medicine and Dentistry, Birmingham B4 6BN, UK; andrea.palermo2004@libero.it

**Keywords:** probiotics, oral health, caries, periodontal disease, halitosis, mucositis, peri-implantitis

## Abstract

**Aim**: Probiotic microorganisms, commonly used to bolster gut health, might also have benefits for dental health, according to certain studies. Probiotics (PBs) are associated with reducing cariogenic pathogens and protecting against periodontal diseases, although the exact way they function in the mouth is not fully clear. Our study aimed to explore the use of PBs to improve oral health, focusing on issues such as cavities, gum disease, bad breath, mucositis, and periimplantitis. **Materials and Methods**: We utilized the Boolean keywords “Probiotics” AND “Oral health” to search the databases of PubMed, Scopus, and Web of Science. The search was restricted to English-language papers published from 1 January 2019 to 13 April 2023. **Results**: A total of 3460 articles were found through our computerized search. After removing duplicates, reviewing the papers, and determining their relevance, 12 were selected for inclusion. **Conclusions**: Assessing how bacteria in food or dietary supplements might alter the stable oral microbiota is a complex task. Although probiotic microorganisms have been found to have proven therapeutic benefits, their application in dental health is not yet solidly backed by evidence. Further research is necessary to thoroughly understand the long-term effects of probiotic bacteria on the oral environment, including their ability to colonize and form biofilms.

## 1. Introduction

Microbes densely and biologically inhabit all body surfaces. Specifically, the oral mucosa is observed to have the second-largest number of host organisms following the colon [1,2,3,4]. While these bacteria are often perceived as potentially hazardous to health [5,6,7], this is not universally true. The risk varies based on the type of microbe and the environment in which the host organism is found [8,9,10,11].

In reality, a substantial relationship exists between the physiological microbiota and the health of the organism it inhabits. This relationship is one of mutual cooperation, where bacteria and yeasts are free to nourish themselves by releasing molecules that are beneficial to the host. They also multiply within certain boundaries, self-regulating to inhibit the growth of more aggressive species and strains [12,13,14].

**Figure 1:**
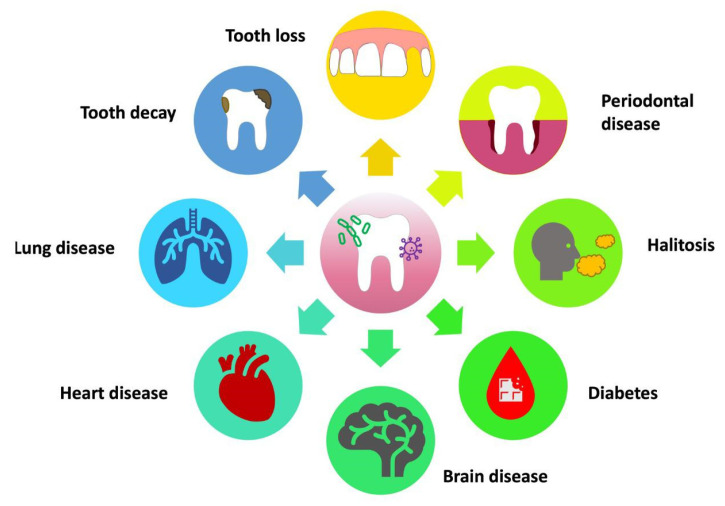
Effects of the imbalance of the oral microbiota on systemic health.

With regard to the oral cavity, it is widely acknowledged that a diverse and healthy microbiota thrives in this area. Many bacterial strains found in the mouth are commensal microorganisms, and they could be beneficial in preventing or treating oral diseases [15]. The concept known as the “ecological plaque theory” argues that the transformation of microbiota from commensal to pathogenic is the result of either synergistic or antagonistic interactions between groups. These interactions change the balance of resident microorganisms, thereby influencing the equilibrium between oral health and the onset of disease [16] (Figure 1).

Physiological microbiota are renowned for safeguarding the oral cavity against infections. In fact, species that are associated with perfect health have been identified, just as various bacterial species connected to oral cavity disorders are acknowledged. The primary role of this set of microbes, known as the buccal bacterial flora, is to inhibit the growth of bacteria and fungi that might infect the mucosa or progress down the oropharyngeal tract to the larynx and beyond [17].

**Figure 2:**
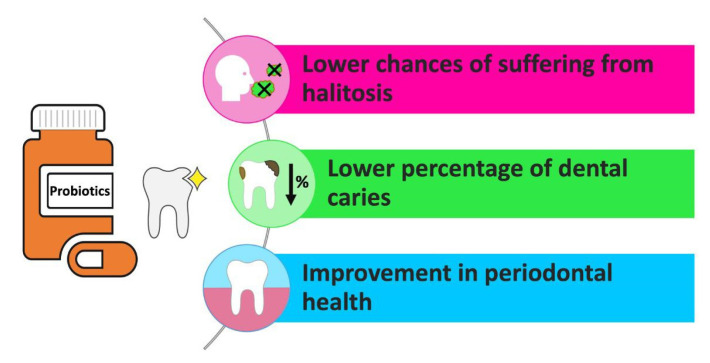
Effects of PBs on oral health.

These bacterial genera are considered a typical element of human microbiota [18] While no species unique to the oral cavity have been found, Lactobacilli generally constitute less than 1% of the total cultivable microbiota in the mouth. Nevertheless, some species are detectable in both fecal and oral samples, including *L. paracasei*, *L. plantarum*, *L. rhamnosus*, and *L. salivarius*, which are often isolated from saliva samples. Research based on culture indicates that Bifidobacteria are among the first anaerobes to colonize the mouth cavity. Breast milk contains both Bifidobacteria and Lactobacilli, suggesting early exposure of the oral cavity to these bacteria. Specific Bifidobacterial species like *B. bifidum, B. dentium*, and *B. longum* have been found in oral samples. Generally considered harmless, Bifidobacteria and Lactobacilli have been linked to health benefits in more fermented food products, dating back to Metchnikoff’s early writings. Regarding physiological microbiota and oral health, variations have been observed in the ability of Lactobacilli isolated from caries-active or healthy individuals to inhibit *Streptococcus mutans* (*S. mutans*) in vitro (Figure 2).

Patients with periodontitis and those with healthy periodontal tissues have been found to possess differing species compositions of the bacteria Lactobacillus and Bifidobacterium in their microbiota. Conversely, dental caries have been associated with both bifidobacteria and lactobacilli. Furthermore, exogenous and opportunistic invaders, possibly introduced through food, have been identified as lactobacilli and bifidobacteria associated with caries [19].

The first scientific investigations into microorganisms and their interactions with humans began in the latter half of the 19th century, albeit from a negative standpoint. As early as 1885, the German pediatrician and bacteriologist Theodor Escherich described the microbiota and the colonization of the infant gastrointestinal tract (GIT), highlighting the beneficial effects of certain bacteria on digestion [20]. It was the German obstetrician Albert Döderlein who first emphasized the positive relationship between vaginal bacteria and lactic acid production, which could suppress or limit pathogenic bacterial growth. Recent studies have validated and expanded on this connection between LAB (lactic acid bacteria) and the human host, a concept that was initially proposed over 100 years ago based on ecological and taxonomic research on the intestine. In 1953, the German physician Werner Georg Kollath coined the term “probiotic expression” to label all organic and inorganic food complexes as “PBs”, contrasting them with “harmful antibiotics”, to classify these food complexes as supplements [21].

PBs, or live microorganisms, can enhance the host’s health when administered in sufficient quantities. Unlike prebiotics, which are non-digestible food components that provide health benefits when utilized by intestinal bacteria, PBs offer benefits such as soluble and insoluble fibers [22]. The precise way PBs operate in the mouth remains unclear. They are linked to the reduction in cariogenic pathogen colony-forming units (CFU) and prevention of periodontal infections, producing substances like lactic acid, hydrogen peroxide, and bacteriocins, and modulating the inflammatory response [23]. Studies largely show their ability to compete with pathogens for adhesion surfaces [24], possibly enhancing oral health indirectly through immunological means rather than directly attacking periodontopathogens. Further studies should delve into the oral microbiota, considering both clinical and immunological aspects. More research into PBs’ underlying immunomodulatory mechanisms in re-instrumentation is needed as this can minimize surgery requirements without completely replacing them.

Furthermore, PBs have been proposed as an effective treatment for candidiasis. Numerous robust studies have demonstrated that PBs, particularly lactobacilli, can inhibit the growth of Candida biofilms in vitro. They have been shown to enhance clinical symptoms, reduce colonization of Candida in various parts of the body, and, in certain cases, reduce the risk of invasive fungal infections in critically ill patients, thus avoiding adverse effects [25]. These benefits have been confirmed in a limited number of clinical trials [26,27].

Similarly, positive effects have been observed with probiotics following antibiotics or chemotherapy treatments. A study by Sharma in 2012 examined the impact of Lactobacillus brevis CD2 lozenges on the frequency and severity of mucositis, as well as tolerance to chemotherapy and radiation therapy [28]. The results also indicated that L. brevis CD2 lozenges reduced the incidence of oral mucositis caused by grade III and IV anticancer therapy, leading to lower overall mucositis rates and higher treatment completion rates [29].

Thus, the potential of probiotics for overall health is significant. This review seeks to emphasize the effects of PBs on oral health and explore the potential mechanisms of probiotic bacteria within the oral cavity.

## 2. Materials and Methods

### 2.1. Protocol and Registration

The PRISMA (Preferred Reporting Items for Systematic Reviews and Meta-Analyses) protocols were followed when conducting this review, and the protocol was registered at PROSPERO under the ID: 451361.

### 2.2. Search Processing

We searched PubMed, Scopus, and Web of Science with a constraint on English-language papers from 1 January 2019 through 13 April 2023 that matched our topic. The following Boolean keywords were utilized in the search strategy: (“Probiotics” AND “Oral health”. These terms were chosen because they best described the goal of our inquiry, which was to learn more about PBs to improve oral health, particularly caries, periodontal disease, halitosis, mucositis, and periimplantitis.

### 2.3. Eligibility Criteria and Study Selection

We chose studies that compared effects determined by use of PBs on various pathologies that may affect the oral cavity. The selection method was divided into two stages: (1) title and abstract evaluation and (2) full-text examination. Any article that met the following criteria was considered: (a) human intervention studies (clinical trials); (b) treatment was compared to other interventions; (c) English language full text. Publications that did not include original data (e.g., meta-analyses, research procedures, conference abstracts, in vitro or animal studies) were excluded. The preliminary search’s titles and abstracts were retrieved and assessed for relevancy. For additional evaluation, full publications from relevant research were obtained. Two separate reviewers (R.S. and C.P.) evaluated the retrieved studies for inclusion using the criteria specified above.

### 2.4. Data Processing

**Figure 3:**
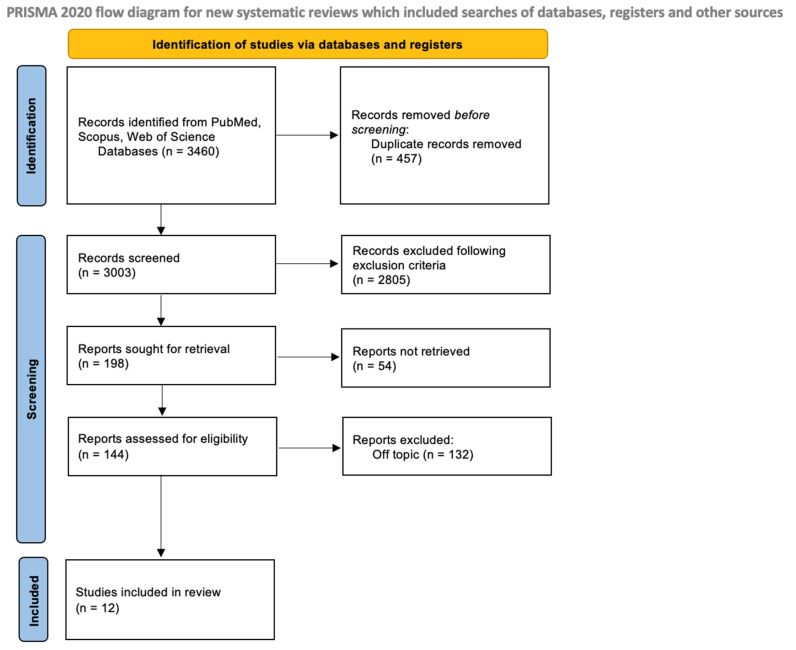
PRISMA flowchart.

Two reviewers (R.S. and C.P.) conducted an independent search of the database to identify relevant studies based on predetermined selection criteria. The quality assessment of the selected articles was also completed independently by the two reviewers. The chosen articles were then saved in Zotero (version 6.0.15). In case of any discrepancies between the two authors, a senior reviewer (F.I.) was consulted to resolve them. The selection process is shown in Figure 3.

### 2.5. Quality Assessment

Using the Cochrane risk-of-bias tool for randomized trials, Version 2, two reviewers evaluated the articles’ bias risk (RoB 2). Any discrepancy was discussed with a third reviewer until an agreement was achieved.

### 2.6. PICOS Criteria

Table 1 depicts the PICOS (Population, Intervention, Comparison, Outcome, Study Design) criteria components, which include population, intervention, comparison, outcomes, and research design and their use in this evaluation.

## 3. Results

The studies discussed in this collection shed light on the potential advantages of probiotics in enhancing oral health across different contexts. Duraisamy et al. (2021) conducted a randomized controlled trial with children, demonstrating that daily probiotic consumption led to a significant reduction in S. mutans levels in saliva [30]. Sarmento et al. (2019) explored the use of probiotic-fortified cheese and its potential to influence the oral microbiota in children [31]. Janiani et al. (2022) found that short-term probiotic milk intake reduced salivary S. mutans levels among children, although long-term effects were not evident [32]. Invernici et al. (2020) focused on patients with periodontitis, suggesting that a specific probiotic could enhance non-surgical periodontal therapy outcomes [33]. Lee et al. (2021) conducted a double-blind study indicating that oral probiotic tablets might contribute to reducing halitosis and improving quality of life related to oral health [34]. Staszczyk et al. (2022) showed that consistent short-term probiotic consumption might slow dental caries onset in children [35]. Laleman et al. (2019) reported that probiotic lozenges significantly reduced pocket depth in patients with periodontitis, particularly in deeper pockets [36]. Santana et al. (2022) explored a multispecies probiotic’s impact on edentulous patients with peri-implant mucositis, revealing reduced bleeding on probing and inflammation [37]. Kang et al. (2020) found that probiotics improved periodontal health in adults, with reduced bleeding and altered oral bacteria levels [38]. Finally, Hasslof et al. (2022) investigated probiotic drops’ potential to prevent dental caries recurrence in preschoolers, with no significant differences observed between the test and control groups [30]. These studies collectively underscore the promising role of probiotics in promoting oral health, although their effectiveness may vary, and long-term impacts require further exploration. The summary of selected records is shown in Table 2.

### Quality Assessment and Risk of Bias

**Figure 4:**
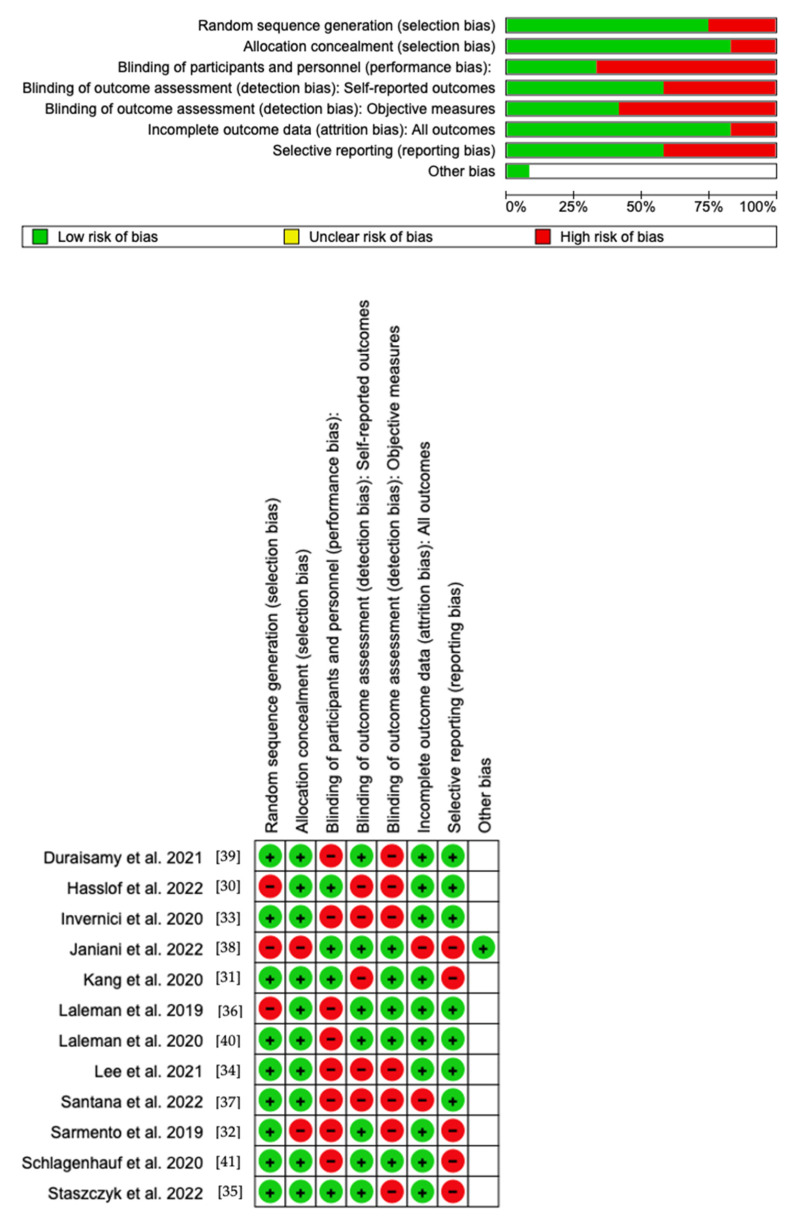
Risk of bias domains of the included studies.

Using RoB 2, the risk of bias was estimated and reported in Figure 4. Regarding the randomization process, 75% of the studies ensured a low risk of bias. However, 25% of the studies excluded performance bias, but 75% reported all outcome data, and 45% of the included studies adequately excluded bias in the selection of reported outcomes, while 55% excluded bias in self-reported outcomes. Overall, all studies were shown to have a low risk of reporting bias. Eight out of twelve studies have a low risk of reporting bias. However, as far as attrition bias is concerned, many studies ensure a low risk of bias.

## 4. Discussion

This section analyzes the observed effects on oral health intake of PBs.

### 4.1. Caries and Associated Microbes

Probiotic bacteria benefit the host in a positive way when ingested appropriately [15]. Intake of PBs is believed to modify the host microflora and preserve or rebuild a natural microbiota. Early oral cavity microbial colonization and maturation are of particular interest in this context because the first one thousand days of life offer a window of opportunity for modifying the microbiota through pre- and probiotic interventions to support normal growth and development [42].

Several studies suggest that consumption of products containing lactobacilli or probiotic bifidobacteria could decrease the quantity of *S. mutans* in saliva [43].

The trend toward a decrease in the number of streptococci in saliva appears to be independent of the product or strain used; however, this effect was not observed in all studies [30].

Hasslof et al. conducted an RCT in order to evaluate the impact of drops containing probiotic bacteria on dental caries recurrence in preschoolers. Further, 38 preschoolers were enrolled after receiving extensive restorative care while sedated or under general anesthesia, and they were monitored again at 6 and 12 months. Parents of kids in the test group were told to put five drops of two strains of Limosilactobacillus reuteri in their children’s mouths at bedtime each day. As a result, they saw that there were no notable variations between the groups [30].

Duraisamy et al. evaluated how well PBs and symbiotics work at reducing the amount of *S. mutans* in children’s saliva following 15 days of daily probiotic and symbiotic curd consumption. Synbiotics are described as “combinations of PBs and prebiotics that have a beneficial effect on the host” by enhancing the survival and implantation of live microbial dietary supplements [22]. The term “prebiotic” was first used by Gibson and Roberfroid to describe a non-digestible food ingredient that has a positive impact on the host by selectively promoting bacterial growth [44]. Children’s salivary *S. mutans* levels were effectively inhibited by PBs and synbiotics. However, probiotic curd was more effective at preventing *S. mutans* growth in children than symbiotic curd [39].

Sarmento et al. in their clinical study tested the effectiveness of cheese with the addition of Lactobacillus casei on the reduction in *S. mutans* and the total number of microorganisms in the volunteers’ saliva after samples were taken at 28 days. The control group was given probiotic-free cheese. The total number of microbes and *S. mutans* in the volunteers’ saliva could be significantly reduced with both products. Nevertheless, only the combination of the product and *L. casei* was able to maintain the low density of *Porphyromonas gingivalis* after treatment while also reducing the density of *Agreggatibacter actinomycetemcomitans.* As a result, the probiotic microorganisms found in the cheese demonstrated their ability to be transported, making it a potential substitute for lowering potentially pathogenic microbiota in the oral cavity [32].

Researchers Janiani et al. looked at how probiotic milk and powder consumption affected children’s plaque scores and salivary levels of *S. mutans*. Indeed, 34 kids between the ages of 3 and 6 were split into three groups; one received probiotic milk, the other received probiotic powder, and the third group served as the control. Saliva samples were examined after 7 days, and it was discovered that consuming probiotic milk and powder resulted in a statistically significant decrease in salivary *S. mutans*. For the group that consumed probiotic powder, the reduction was greatest. However, only drinking probiotic milk significantly decreased plaque scores. Probiotic use over the long term may have a karyostatic effect, but more studies are necessary to confirm this [38].

Staszczyk et al. conducted a clinical trial that sought to ascertain whether daily intake of chewing gums containing *Lactobacillus salivarius HM-6 Paradens* was associated with a reduction in caries (tooth decay) in preschoolers with high caries levels when compared to a control group receiving standard care. The study discovered a significant decline in the incidence and prevalence of early childhood caries after the 1-year follow-up period, indicating that *Lactobacillus salivarius HM-6 Paradens* may be more effective in secondary prevention (slowing the progression of existing lesions) than in primary prevention (preventing new lesions), but additional research is required to confirm these findings and investigate long-term effects. While the probiotic group maintained their plaque scores more consistently throughout the trial period, the study did not demonstrate a substantial reduction in plaque buildup [35].

Unfortunately, with regard to dental caries, the groups were relatively small and the studies rather short [30].

Additionally, it is crucial to realize that the salivary level of caries-associated microbes is not directly related to dental caries itself. Therefore, no conclusive statement can be made about the effects of probiotic bacteria on dental caries [45].

### 4.2. Periodontal Disease

*Lactobacillus reuteri* probiotic is revealed to be a useful instrument for gingivitis and periodontal disease [46].

Schlagenhauf et al. evaluated the consumption of probiotic-containing Lactobacillus reuteri beneficial for periodontal health with an improvement in all parameters of BOP, GI, PI, attachment level, and pocket depth in a group of navy sailors [41].

A randomized trial of Kang et al. analyzes the effects of *W. cibaria CMU*. It is a Gram-positive bacterium capable of transforming glucan of S. mutans into water-soluble dextran with the inhibition of carious disease. The effect of this probiotic is also efficacious against *F. nucleatum* in the therapy of periodontal disease [31].

Invernici et al. examined the effects of *Bifidobacterium animalis subsp. lactis HN019* (HN019) on periodontal clinical parameters, including gingival bleeding, presence of plaque, gingival tissue immunocompetence, including expression of beta-defensin (BD)-3, toll-like receptor 4 (TLR4), differentiation cluster (CD)-57 and CD-4, and saliva immunological properties, including IgA levels. HN019’s antimicrobial qualities and buccal epithelial cell adhesion (BEC) were also investigated. It was discovered that *B. lactis HN019* is a potential probiotic to use in nonsurgical periodontal treatment of patients due to its antimicrobial and immunological properties [33].

The article of Laleman et al. explores the demand for new treatments to lessen the risk of tooth loss and periodontal disease development posed by residual pockets. The adjunctive impact of a dual-strain *L. reuteri* probiotic on the re-instrumentation of residual pockets is the study’s focus. According to the findings, re-instrumentation with ultrasonic tips combined with *L. reuteri* PBs increases PPD statistically significantly when compared to control groups. The PPD levels were lower, the pockets were better closed, there were fewer pockets that needed surgery, and the risk of disease progression was lower in the probiotic group. PBs may have beneficial impacts on oral health through immunological processes rather than by directly suppressing periodontopathogens. Future studies should analyze the oral microbiota more thoroughly and look at immunological indicators in addition to clinical characteristics. The underlying immunomodulatory mechanisms of PBs in re-instrumentation require more study. Although re-instrumentation alone cannot completely replace surgery, it can lessen the amount of surgery that patients require [40].

### 4.3. Halitosis

*W. cibaria*, an oral probiotic, is evaluated by Dong-Suk Lee et al. for its effects on halitosis. Random assignment was used to place participants in either the experimental or control groups. For eight weeks, they consumed either *W. cibaria* or a placebo before going to bed, depending on which group they belonged to. Subjective halitosis, subjective oral health status, depression, self-esteem, and oral-health-related quality of life were the indicators that were measured. At baseline and eight weeks later, measurements were taken. Participants displayed statistically significant variations in subjective halitosis and quality of life associated with oral health [34].

### 4.4. Mucositis and Peri-Implantitis

Santana et al. conducted a double-blind, randomized, controlled trial that investigated the effects of using multispecies PBs as an adjuvant therapy for the treatment of peri-implant mucositis (PiM). The study compared the outcomes of probiotic therapy combined with conventional mechanical debridement (MD) to MD alone. This study introduced a cocktail of microorganisms combining *Bifidobacterium* and *Lactobacillus* strains as a potential treatment for PiM. The probiotic therapy provided additional clinical and immunological benefits when used alongside MD. However, the study acknowledged the limitation of its short assessment period and recommended further research to evaluate the long-term effects of probiotic therapy for PiM treatment. The specific bacterial strains used in probiotic formulations and their interactions with the oral microbiota are believed to contribute to the variability in outcomes. The researchers suggested that the immunomodulatory and antimicrobial properties of PBs could explain these positive outcomes [37].

Laleman et al. conducted a study that looked at how PBs affected the non-surgical management of peri-implantitis, an infection that develops around dental implants. The study’s findings indicated that non-surgical therapy of peri-implantitis resulted in a considerable improvement in clinical characteristics. Except for a greater reduction in the PI at the peri-implantitis locations in the probiotic group compared to the control group, the study was unable to show the added utility of PBs in this therapy. An altered sensation in the oral cavity was the sole negative effect noted, but it had nothing to do with the actual study itself. The study found that non-surgical debridement and oral hygiene recommendations improved the clinical traits of peri-implantitis locations in a statistically significant manner. However, attaining entirely healthy peri-implant tissue was difficult, indicating that non-surgical treatment might not be enough to eliminate peri-implant inflammation. The essay recommends two areas of potential future study. The first is the use of better tools, including titanium tips for ultrasonic debridement, and the recurrent use of probiotic drops to increase local contact time. Second, after the surgical phase, research the use of PBs to maintain peri-implant stability. Peri-implantitis can be treated non-surgically as a first line of treatment. The report indicates that, to better understand the underlying mechanisms of healing and enhance therapy, future research should not only concentrate on clinical and microbiological aspects but also investigate inflammatory markers. As regulation of the inflammatory response is thought to be a potential probiotic action mechanism, monitoring inflammatory markers is advised while researching PBs as a therapeutic [36].

Recalling that the studies take into account various PBs or the same PBs but in various combinations, as well as the fact that the patient groups range in terms of age, socio-cultural traits, and comorbidities, it is important to critically evaluate the research findings.

The possible advantages of any bacterial strain for dental health should be carefully considered. Additionally, a mouth bacterium should not necessarily be regarded as a probiotic [34].

Numerous medicinal advantages come from probiotic microorganisms: they may help lessen bad breath, strengthen the local immune system, boost defense against possible oral infections, and assist maintaining a healthy balance of the bacteria in the mouth that cause tooth decay and gum disease. To elucidate how probiotic bacteria affect the resident flora, further studies are needed to better understand the long-term effects of probiotic bacteria on the oral cavity and their ability to colonize and create biofilms [15]. The studies are heterogeneous in the population, and it is, moreover, difficult to predict how many bacteria present in foods or food supplements will affect the oral microbiota [42].

## 5. Conclusions

It is essential to closely analyze a number of factors for a proper evaluation of the data acquired in the conducted investigations. It is important to keep in mind that these investigations include several bacterial strains (BS) or the same strains but in different combinations. It is crucial to remember that the patient populations included show considerable differences, such as differences in age, socio-cultural traits, and comorbidities. These elements may have a significant impact on the study results and how applicable they are to clinical practice. It is important to note that each bacterial strain has the potential to promote oral health, but, in order to properly understand their effect, it is important to evaluate each one separately. It is important to recognize that not all bacteria present in the mouth may be categorized as probiotics.

A variety of therapeutic advantages of probiotic bacteria have been proven. According to certain studies, they may help prevent halitosis, maintain a healthy balance of the oral bacterial flora, and prevent the growth of dangerous bacteria that cause tooth decay and gum disease. Additionally, they might strengthen the neighborhood immune system, boosting its defenses against any mouth infections. Given these benefits, there is no justification for banning their use as they may help to maintain excellent dental health.

However, it is crucial to stress that further research is required to fully comprehend probiotic bacteria’s persistent action in the oral cavity and their ability to colonize and create biofilms. This will make it easier to understand how they affect the local flora and to spot any obstacles or limits to their effectiveness.

## Figures and Tables

**Table 1:** PICOS criteria.

Criteria	Application in the Present Study
Population	Both children and adults
Intervention	Use of PBs to improve oral health
Comparisons	Comparing effect of use of PBs on different oral pathologies
Outcomes	Efficacy in preventing caries, periodontal disease, halitosis, mucositis, and periimplantitis
Study design	Clinical Trials.

**Table 2:** Descriptive summary of item selection.

Authors	Type of Study	Object	Study Design and Timeline	Results	Number of Participants	nn
Duraisamy et al., 2021 [39]	Randomized controlled trial	After 15 days of daily consumption of probiotic and synbiotic curd, this study seeks to determine how well PBs and synbiotics inhibit the level of *S. mutans* in children’s saliva.	40 children aged 6–12 received probiotic and synbiotic curd for 15 days, saliva samples collected, and *S. mutans* levels estimated.	Both groups showed significant decrease in salivary *S. mutans* counts, with probiotic group showing higher growth inhibition.		
Sarmento et al., 2019 [32]	Clinical study	Evaluate the impact of petit-suisse cheese added with PBs on the salivary microbiota of children	Administration of cheese fortified with *L. casei* for 28 days and subsequent evaluation of saliva	The probiotic microorganisms that can be carried by the petit-suisse cheese have been developed, offering a potential substitute for reducing potentially harmful microbiota in the mouth		
Janiani et al., 2022 [38]	Randomized controlled trial	To investigate the impact of a brief intake of probiotic milk on children’s plaque scores and salivary number of *S. mutans*	Administration of PBs for one week to 34 children aged 3 to 6 years, final comparison with control group	There was a very important reduction in *S. mutans* in saliva with karyostatic effect after probiotic intake, but no known long-term effects	34	
Invernici et al., 2020 [33]	Randomized clinical trial	Evaluate the effects of *Bifidobacterium animalis* subsp. *lactis HN019* on clinical periodontal parameters, immunocompetence, and saliva immunological properties.	Scaling and root planing (SRP) was performed on thirty patients, and they were observed at the beginning, at 30, and at 90 days. Probiotic lozenges were administered to the participants for 30 days in either the Test or Control groups.	The probiotic *B. lactis* HN019 may enhance the results of non-surgical periodontal therapy	30	
Lee et al., 2021 [34]	Randomized, Double-Blind, Placebo-Controlled Study	The purpose of this research was to determine how taking tablets of the oral probiotic *Weissella cibaria* (*W. cibaria*) affected psychosocial indicators and halitosis.	Random selection was used to place the participants in either the experimental or control groups. Depending on which group they belonged to, they consumed *W. cibaria* CMU or a placebo just before going to sleep every day for eight weeks.	For eight weeks, taking the oral probiotic could be a helpful nursing intervention for halitosis reduction and quality-of-life enhancement in relation to oral health	100	
Staszczyk et al., 2022 [35]	Open Label Randomized Controlled Trial	Determine if chewing tablets containing thermally inactivated *L. salivarius* decreased the 12-month caries increase relative to the control group after two weeks of daily ingestion.	A study involving 140 healthy children aged 3–6 with or without ECC was conducted. The primary end measure was the 1-year increase in dental caries incidence and prevalence, while secondary outcomes included cavitated and apparent dentinal caries and dental plaque buildup	The probiotic group’s initial and end mean OHI-S scores did not significantly differ from one another. In conclusion, consistent short-term consumption of PBs may slow the onset of caries.	140	
Laleman et al., 2020 [40]	Randomized controlled clinical trial	To investigate the supplemental impact of a probiotic *Lactobacillus reuteri* strain on the re-instrumentation of residual pockets.	39 periodontitis patients underwent re-instrumentation, probiotic or placebo drops administered, and lozenges for 12 weeks. Examined probing pocket depth, recession, bleeding on probing, and plaque levels	Probiotic lozenges significantly reduced overall PPD after 24 weeks, especially in intermediate and deep pockets, with fewer surgically necessary sites and pockets. The group reduced thickness from 4 mm to 3 mm at 24 weeks.	39	
Santana et al., 2022 [37]	Randomized controlled trial	Check the effects of a multispecies probiotic supplemented with mechanical debridement (MD) on changes in BOP in edentulous patients with peri-implant mucositis (PiM). The supplement contains *Lactobacillus rhamnosus HN001TM*, *Lactobacillus paracasei Lpc-37*^®^, and *Bifidobacterium animalis* subsp. *lactis HN019TM*.	Patients were randomly assigned to probiotic test or placebo control groups. MD and topical gel applications were applied twice daily for 12 weeks. Clinical and immunological measurements were taken at baseline, 12, and 24 weeks. Statistical analysis was used.		36	
Laleman et al., 2019 [36]	Randomized pilot study	Analyze the clinical and microbiological advantages of a dual-strain probiotic of *L. reuteri* for the non-surgical treatment of first peri-implantitis.	Patients with peri-implantitis underwent full-mouth prophylaxis and cleaned sites. Study lozenges and drops were applied to peri-implantitis areas, with probiotics and placebos given. Implant-level variables, bleeding, PPD, full-mouth bleeding, plaque scores, and subgingival, tongue, and saliva samples were analyzed for microbes.	statistically significant difference. After 12 and 24 weeks, clinical measures showed significant decreases. The probiotic group experienced a greater decline in plaque levels at implant level, while the probiotic group had a larger reduction in full-mouth BOP sites. No measurable microbiological changes were observed.	10	
Schlagenhauf et al., 2020 [41]	Randomized controlled trial	The purpose of this trial was to establish if the regular consumption of *L. reuteri* PBs can help periodontal health and oral health in navy sailors.	A 42-day study involved 72 healthy sailors in two groups: the test group, who consumed probiotic strains of *L. reuteri* twice daily, and the placebo group, who received no PBs. Primary outcome was bone marrow opacity.	Probiotic *L. reuteri* strains consumption significantly improved test group scores at 14 and 42 days, proving a practical and easy method for maintaining periodontal health and oral care.	72	
Kang et al., 2020 [31]	Randomized, double-blind, placebo-controlled trial	The aim of this study was to evaluate the effects of W. cibaria CMU (oraCMU)on periodontal health and oral microbiota	92 adults (20–39 years old) without periodontitis underwent dental scaling and root planing and were randomized to either the probiotic or placebo groups. When taken once daily for eight weeks, the 800 mg probiotic tablet delivered 1.0 108 CFU/g of W. cibaria CMU (oraCMU). BOP, PD, GI, plaque index (PI), and microbiota in the gingival sulcus were all examined as periodontal clinical parameters.	Over an 8-week period, BOP improved more in the probiotic group. During the intervention, no discernible inter-group differences in PD, GI, or PI were found. The probiotic group was found to have fewer oral bacteria. At 8 weeks, there was a significant difference between the two groups in the levels of *Fusobacterium nucleatum* (*F. Nucleatum*) and *Staphylococcus aureus.* For patients with periodontitis, CMU enhances BOP and the oral environment.	92	
Hasslof et al., 2022 [30]	Randomized controlled trial	To assess the impact of drops containing probiotic bacteria on dental caries recurrence in preschoolers	38 preschoolers were enrolled after receiving extensive restorative care while sedated or under general anesthesia, and they were monitored again at 6 and 12 months. Parents of kids in the test group were told to put 5 drops of two strains of *L. reuteri* in their children’s mouths at bedtime each day.	There were no notable variations between the groups.	38	

## Data Availability

Data sharing is not applicable.

## Abbreviations

*B. Bifidum*	Bifidobacterium bifidum
*B. Dentium*	Bifidobacterium dentium
BEC	buccal epiteliale Cell adhesion
*B. Longum*	Bifidobacterium longum
CFU	Colony-forming units
CI	confidence interval
ECC	Early Childhood Caries
F.	Nucleatum Fusobacterium Nucleatum
GI	gingival index
GIT	Infant gastrointestinal tract
ICDAS II	International Caries Detection and Assessment System II
IL	interleukin
LAB	Lactic Acid bacteria
*L. brevis* CD2	Lactobacillus brevis CD2
*L. paracasei*	Lacticaseibacillus paracasei
*L. plantarum*	Lactobacillus plantarum
*L. reuteri*	Lactobacillus Reuteri
*L. rhamnosus*	Lacticaseibacillus rhamnosus
*L. salivarius*	Ligilactobacillus salivarius
MD	mechanical debridement
mPI	modified plaque index
mSBI	modified sulcus bleeding index
OHI-S index	Simplified Oral Hygiene Index
PBs	Probiotics
PI	plaque index
PiM	peri-implant mucositis
PPD	pocket probing depth
PD	probing depth
*S. mutans*	Streptococcus mutans
SRP	Scaling and root planing
TNF	tumor necrosis factor
*W. cibaria*	Weissella cibaria
